# Bariatric Surgery and Non-Alcoholic Fatty Liver Disease: Current and Potential Future Treatments

**DOI:** 10.3389/fendo.2014.00164

**Published:** 2014-10-27

**Authors:** Akira Sasaki, Hiroyuki Nitta, Koki Otsuka, Akira Umemura, Shigeaki Baba, Toru Obuchi, Go Wakabayashi

**Affiliations:** ^1^Department of Surgery, Iwate Medical University School of Medicine, Morioka, Japan

**Keywords:** non-alcoholic fatty liver disease, non-alcoholic steatohepatitis, obesity, diabetes, bariatric surgery, laparoscopy

## Abstract

Non-alcoholic fatty liver disease (NAFLD) and non-alcoholic steatohepatitis (NASH) are increasingly common cause of chronic liver disease worldwide. The diagnosis of NASH is challenging as most affected patients are symptom-free and the role of routine screening is not clearly established. Most patients with severe obesity who undergo bariatric surgery have NAFLD, which is associated insulin resistance, type 2 diabetes mellitus (T2DM), hypertension, and obesity-related dyslipidemia. The effective treatment for NAFLD is weight reduction through lifestyle modifications, antiobesity medication, or bariatric surgery. Among these treatments, bariatric surgery is the most reliable method for achieving substantial, sustained weight loss. This procedure is safe when performed by a skilled surgeon, and the benefits include reduced weight, improved quality of life, decreased obesity-related comorbidities, and increased life expectancy. Further research is urgently needed to determine the best use of bariatric surgery with NAFLD patients at high risk of developing liver cirrhosis and its role in modulating complications of NAFLD, such as T2DM and cardiovascular disease. The current evidence suggests that bariatric surgery for patients with severe obesity decreases the grade of steatosis, hepatic inflammation, and fibrosis. However, further long-term studies are required to confirm the true effects before recommending bariatric surgery as a potential treatment for NASH.

## Introduction

In recent years, the obese population has been rapidly increasing because of increases in diets rich in saturated fat and processed carbohydrates and sedentary lifestyles. The metabolic syndromes that are considered risk factors of arteriosclerotic diseases, such as abnormal glucose tolerance, hyperlipemia, and hypertension, have increased alongside obesity in the developed world (1). Non-alcoholic fatty liver disease (NAFLD) is a liver phenotype of metabolic syndrome. Factors that affect the morbidity of this disease include genetic background, an epigenetic control mechanism, the fat toxicity of free fatty acid, and a natural immunity system of intestinal bacteria. Based on current trends, the rate of obesity in the US is projected to reach approximately 40% by 2025 (2). However, the prevalence of NAFLD is not well known, though it is estimated at 6.3–33% worldwide. In North America, the prevalence of NAFLD is estimated at about 20%, and the prevalence of its more progressive subtype, non-alcoholic steatohepatitis (NASH), is approximately 2–3% (3–6).

Non-alcoholic fatty liver disease is the most common cause of liver dysfunction worldwide, and obesity is a well-documented risk factor for the disease. It is highest in populations with preexisting metabolic conditions, such as obesity, T2DM, and dyslipidemia (7). NASH is often an incidental diagnosis and can be associated with other disease processes, such as hypopituitarism, hypogonadism, hypothyroidism, polycystic ovarian syndrome, and obstructive sleep apnea syndrome. It is also frequently associated with pancreatoduodenal resections (8).

Perioperative liver biopsy in patients with severe obesity can result in complications, such as bleeding and sampling error, making exact diagnosis difficult to achieve. Among patients with severe obesity undergoing bariatric surgery, the prevalence of NAFLD can exceed 90%, and up to 5% of these patients may have unsuspected cirrhosis. Bariatric surgery can improve obesity-related diseases in addition to reducing body weight; as a result, it has more recently been termed “metabolic surgery.”

We conducted a literature review using the Medline database. We identified studies conducted between 1995 and 2014 that examined the impact of bariatric surgery on NAFLD. This review evaluates the benefits and potential treatment option of bariatric surgical procedures for NAFLD as described in the literature.

## Medical Treatment of NAFLD

Past studies investigating lifestyle changes, such as diet, exercise, and behavior modification that resulted in weight loss of 5–10%, have shown that these changes can improve steatosis in some patients (7, 9). Greater weight loss of at least 10% has also been shown to improve inflammation. However, it is important to note that adherence to lifestyle interventions can be problematic. In several studies, only 15% of patients achieved a weight loss >10%, and most of these people regained the weight. The best average weight loss among participants has been reported at 3–4 kg at 2 and 4 years (10).

Although no drugs are specifically licensed for the treatment of NAFLD, there is evidence to support the use of selected agents. Antiobesity medications such as orlistat, and drugs that augment insulin sensitivity and reduce plasma glucose concentrations and oxidative stress such as thiazolidinediones and metformin are among those that have been shown to improve liver histology in NAFLD (11–14). Orlistat inhibits enteric lipid absorption and has been promoted as a weight loss aid. It has also been evaluated as a potential therapy for NASH (15).

It is thought that GLP-1 receptor agonists work toward improvement of insulin resistance because not only can they improve the effect of insulin secretion but can also promote glucose uptake by the liver, adipose tissue, and skeletal muscle (16). Many studies have reported improvement outcomes with GLP-1 receptor agonist therapy in animal NAFLD models and NAFLD patients, and the expectation of this drug’s effectiveness to treat NAFLD is increasing (17–19). According to a recent systematic review and meta-analysis, GLP-1 receptor agonists significantly decreased the weight of obese patients regardless of the existence of T2DM (20). Astrup et al. planned a clinical study of the GLP-1 receptor agonists to treat severely obese patients without T2DM. These researchers reported that weight loss was significantly greater in the patients treated with GLP-1 receptor agonists than in the patients with only diet and exercise modifications. These results showed better outcomes than in the patients with orlistat as a comparative drug (21). Thus, the use of pharmacological agents for obesity is quickly gaining popularity for patients with mild obesity and visceral adipose obesity. Incretin mimetics may therefore represent a novel therapeutic option in the future for slowing the progression of NAFLD. However, larger studies with longer treatment durations are required to confirm whether incretin mimetics confers any benefits above their effects on weight loss.

## Bariatric Surgery and NAFLD

The goal of bariatric surgery is not only to achieve satisfactory weight loss but also to obtain improvements in obesity-related comorbidities, including T2DM, obstructive sleep apnea syndrome, hyperlipidemia, and hypertension. However, no randomized controlled trials examining the effects of bariatric surgery on NAFLD were found in the literature search. Two meta-analyses evaluated the effect of bariatric surgery on the liver histology of patients with NAFLD (22, 23). Mummadi et al. reported that the improvement or resolution rates of steatosis, steatohepatitis, and fibrosis of 15 studies and 766 paired liver biopsies after bariatric surgery were 91.6, 81.3, and 65.5%, respectively (22). Complete resolution of NASH was archived in 69.5% of patients. However, a recently published Cochrane review concluded that the lack of randomized clinical trials or quasi-randomized clinical studies has prevented a definitive assessment of the benefits and harms of bariatric surgery as a therapeutic approach for patients with NASH (23).

## Roux-En-Y Gastric Bypass

Bariatric surgery has a positive effect on obesity-related diseases in addition to reducing body weight. Because of this, it has been more recently termed metabolic surgery. Roux-en-Y gastric bypass (RYGB) has a reportedly greater positive effect on T2DM than restrictive procedures. This mechanism is not understood; however, two theories exist: the hindgut and foregut hypotheses. In the hindgut hypothesis, bypass of the upper small intestine diverts food directly to the lower small intestine, where glucagon-like peptide-1 (GLP-1) secretory cells promote GLP-1 secretion. In the foregut hypothesis, an unknown glucose-tolerance-complicating factor that inhibits GLP-1 secretion is discharged from the upper small intestine; because bypass surgery prevents food from passing through the upper small intestine, this factor is no longer secreted (24, 25). Post-operatively, patients who undergo RYGB have shown a marked reduction in hepatic lipid content and improved hepatic insulin sensitivity well before significant weight loss occurs. These benefits to the liver are directly related to at least two enteroendocrine cells-synthesized gut peptides GLP-1 and peptide YY (26–29).

No randomized clinical trials of bariatric surgery in NAFLD were found in the literature. Fourteen studies including reviews showed that RYGB has been associated with improvement in NAFLD (11, 28, 30–42) (Table 1). Overall, RYGB in obese patients has decreased the grade of steatosis, hepatic inflammation, and fibrosis. However, some studies reported a few patients with worsening or new fibrosis (30, 32, 33, 35, 37).

**Table 1:** **Effect of RYGB on NAFLD**.

Reference	Patients	Types of study	Main outcomes (improvement)	Follow-up (months)
Silverman et al. (30)	91	Retro	Steatosis and fibrosis	18.4
Mattar et al. (31)	70	Pros	Steatosis and fibrosis	15
Clark et al. (32)	16	Pros	Steatosis, inflammation, and fibrosis	0.8
Mottin et al. (33)	90	Retro	Steatosis (82%)	12
Klein et al. (34)	7	Pros	Fibrosis and inflammation	12
Barker et al. (28)	19	Pros	Steatosis, inflammation, and fibrosis	21.4
Csendes et al. (35)	16	Pros	Histology (80%)	22
de Almeida et al. (36)	16	Pros	Steatosis, inflammation, and fibrosis	23.5
Furuya et al. (37)	18	Pros	Steatosis and fibrosis	24
Liu et al. (38)	39	Retro	Steatosis, inflammation, and fibrosis	18
Weiner et al. (39)	116	Retro	Complete regression (83%)	18.6
Meretto et al. (40)	78	Retro	Resolved fibrosis (50%)	Unavailable
Vargas et al. (41)	26	Pros	Steatosis, inflammation, and fibrosis	16
Tai et al. (42)	21	Pros	Steatosis, inflammation, and fibrosis	12

*Pros, prospective; Retro, retrospective*.

## Sleeve Gastrectomy and Gastric Banding

Recent studies have shown that laparoscopic sleeve gastrectomy (LSG) is safe and effective, resulting in weight loss somewhere between the rates of laparoscopic RYGB and laparoscopic adjustable gastric banding (LAGB) (43, 44). Although LSG is a restrictive procedure, the removal of the gastric fundus, the primary site of ghrelin production, appears to have a hormonal effect that enhances weight loss by reducing appetite (25).

Based on questionnaire survey results from 50 countries, Buchwald et al. reported that 340,768 bariatric surgeries were performed worldwide in 2011 (45), which included 47% LRYGB, 28% LSG, and 18% LAGB. LSG, an initial bariatric surgery for severely obese patients, is a technique used to lower the rates of complication and surgical death. For these reasons, LSG has increased rapidly worldwide and is predicted to become the most frequently performed bariatric surgery.

Only four studies were found that utilized LAGB or LSG as a restrictive procedure to examine the effects on NAFLD (46–49) (Table 2). Dixon et al. conducted paired liver biopsies on 36 obese patients – the first at the time of LAGB and the second after weight loss. On follow-up biopsies at 25.6 months after LAGB, the mean percentage of excess weight loss was 52%, and there were major improvements in lobular steatosis, necroinflammatory changes, and fibrosis. However, portal abnormalities remained unchanged (46). The same author examined a second similar investigation of 60 severely obese patients after LAGB. On follow-up biopsies at 29.5 months after LAGB, only 10% displayed NASH. Improvements in steatosis, inflammation, and fibrosis, as well as in biochemical markers of liver function, were seen in all of the patients. The researchers concluded that decreases in gamma-glutamyl transferase concentrations were associated with histological improvement (47). Mathurin et al. reported no significant differences among LAGB, RYGB, and biliointestinal bypass groups, and those who progressed to fibrosis became more insulin resistant. Five years after bariatric surgery for severe obesity, almost all of the patients had low levels of NAFLD, whereas fibrosis slightly increased (48).

**Table 2:** **Effect of restrictive bariatric surgery on NAFLD**.

Reference	Patients	Types of surgery	Main outcomes (improvement)	Follow-up (months)
Dixon et al. (46)	36	LAGB	Steatosis, inflammation, and fibrosis	25.6
Dixon et al. (47)	60	LAGB	Steatosis, inflammation, and fibrosis	29.5
Mathurin et al. (48)	381	LAGB	Steatosis	50
Karcz et al. (49)	236	LSG	AST, ALT, triglyceride and HDL levels	12

*Pros, prospective; Retro, retrospective*.

Studies of follow-up biopsies for NAFLD in LSG were not found in the literature review. Karcz et al. reported on the effect on NASH diagnosed at the time of LSG and NASH-related comorbidities using clinical and biological data at 1- and 3-year follow-ups. A significant improvement of AST, ALT, triglyceride, and HDL levels was shown in the 87 NASH patients (49).

As a mechanism that improves NAFLD, GLP-1 plays an important role after LSG. It has been shown that GLP-1 receptor agonists may be an effective tool for the treatment of NASH. This is due to the continuous increase of serum GLP-1 levels and the reduction of hepatic glucose production, which are expected indirect actions of GLP-1 after LSG. However, a future study is required to confirm this. Umemura et al. reported that the mean weight reduction was 11 kg at 1 month after LSG for patients with severe obesity; the mean GLP-1 levels increased, while mean ghrelin levels decreased significantly (50). The researchers also reported that the changes in these hormones might have derived from a resection of the gastric fundus and the hindgut effect with accelerated gastric emptying.

## Biliopancreatic Diversion Procedure

Only two studies were found the biliopancreatic diversion procedures to evaluate the effects on NAFLD (51, 52). Keshishian et al. studied repeat liver biopsies on 78 patients after a duodenal switch procedure. Hepatic inflammation slightly worsened at 6 months, but improvements were seen at and beyond 12 months. By 36 months, the histological degree of steatosis had improved by 60% and the severity of inflammation improved by three grades (51). Kral et al. studied liver biopsies on 104 patients after a biliopancreatic diversion procedure. Steatosis grades decreased in correlation with weight loss as expected, but the researcher observed a post-operative increase in fibrosis in 40% of the patients, a decrease in 27%, and no change in 33% (52). The researcher suggested that several factors such as alcohol, bacterial products, iron storage, deficiencies of antioxidants, protein, lipotropic factors, or the generation of reactive oxygen species might explain the development or lack of improvement in a few patients with severe fibrosis or cirrhosis.

## Bariatric Surgery as a Potential Treatment for NAFLD

The current hypothesis for the development of NAFLD is that obesity and insulin resistance increase the release of free fatty acids from adipocytes (53), and hepatic insulin resistance and hepatic steatosis precede the development of T2DM (54).

Bile acids are important regulators of energy balance through the unclear receptor farmesoid X receptor (FXR) and the G-protein-coupled membrane receptor TGR5. Activation of FXR by bile acids after a meal induces synthesis of the intestinal peptide hormone FGF-19 and triggers a cascade that controls fed-fasted state metabolism. Bile acids could also promote weight loss by increasing energy expenditure in brown adipose tissue via TGR5. Bile acids might be involved in bariatric surgery-induced changes in energy homeostasis through two mechanisms: increased secretion of bile acids with direct effects on energy balance and increased delivery to the distal intestine due to nutrient and bile re-routing, with increased stimulation of L-cell production and the release of hormones with attenuating effects on bariatric surgery (55). Patti et al. reported that total serum bile acid concentrations were higher in RYGB patients than in obese patients (56). Haluzíková et al. reported that the effects of LSG on serum concentrations of FGF-19 and FGF-21 along with circulating bile acids and other relevant hormonal and biochemical parameters (57). The researchers concluded that increased FGF-19 and decreased ghrelin concentrations could have partially contributed to the improvement of systemic inflammation and some metabolic parameters after LSG, while changes of FGF-21 are rather secondary because of weight loss. Pournaras et al. reported that gastric bypass leads to increased plasma bile acids, FGF-19, incretin, and satiety gut hormone concentrations (58).

In a recent systematic review, post-operative resolution or improvement of T2DM occurred in 73% of patients (59). Potential mechanisms of T2DM remission underlying the direct anti-diabetic impact of bariatric surgery include enhanced nutrient stimulation of GLP-1, altered physiology from excluding ingested nutrients from the upper intestine, compromised ghrelin secretion, improved hepatic insulin sensitivity, and improved peripheral insulin sensitivity. The changes in the rate of eating, gastric emptying, intestinal transit time, nutrient absorption, and sensing, as well as bile acid metabolism, may also be implicated (60, 61). Bariatric surgery, which offers the effects of metabolic surgery, should be considered for T2DM patients having difficulty continuing with medical treatment and a potential for future deterioration and diabetic complications.

**Figure 1:**
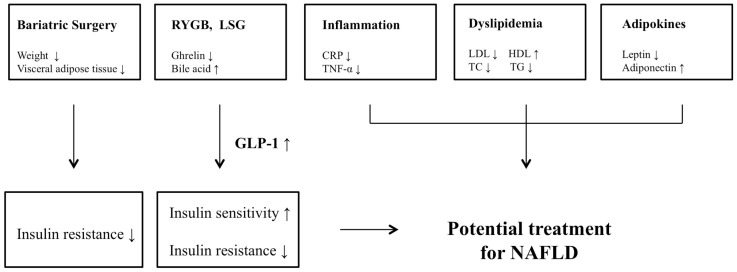
**Potential mechanisms or improvement for NAFLD after bariatric surgery**.

The mechanism of how bariatric surgery plays a role as a potential treatment for NAFLD is also complex and not fully understood. Bariatric surgery is likely to have potential benefits in ameliorating the factors such as insulin resistance, lipid profile, inflammation, weight loss, and adipokines that contribute in a marked way to the pathogenesis of NAFLD (Figure 1).

## Current Guidelines

Currently, the only proven effective treatment for NAFLD is weight loss. Many reports have shown that when performed by a skilled surgeon, bariatric surgery is safe and effective for reducing weight, improving quality of life, decreasing obesity-related diseases, and increasing life expectancy. However, there is a lack of randomized controlled trials examining the effects of bariatric surgery on NAFLD; the only available studies are either retrospective or prospective cohort studies (5).

The American Association for the Study of Liver Diseases (AASLD), American College of Gastroenterology (AGG), and American Gastroenterological Association (AGA) recommend that foregut bariatric surgery should not be contraindicated in otherwise eligible obese individuals with NAFLD or NASH. However, these organizations recognize that it is premature to consider foregut bariatric surgery as an established option to treat NASH (5).

## Conclusion

The current evidence suggests that bariatric surgery for patients with severe obesity decreases the grade of steatosis, hepatic inflammation, and fibrosis. However, the lack of randomized clinical trials demonstrating the beneficial effects of bariatric surgery procedures for treatment of NAFLD prevented us from reaching a scientifically sustained conclusion. Positive results have been observed in cohort studies; however, this research has a high risk of bias. The research also reported a potential risk for worsening fibrosis scores. Therefore, bariatric surgery must be assessed in randomized clinical trials.

The mechanism of bariatric surgery’s role as a potential treatment for NASH is complex and not fully understood. Further long-term studies are required to confirm the true effects before recommending bariatric surgery as a treatment option for NASH.

## Conflict of Interest Statement

The authors declare that the research was conducted in the absence of any commercial or financial relationships that could be construed as a potential conflict of interest.
